# Role of Fluid and Sodium Retention in Experimental Ventilator-Induced Lung Injury

**DOI:** 10.3389/fphys.2021.743153

**Published:** 2021-09-13

**Authors:** Simone Gattarello, Iacopo Pasticci, Mattia Busana, Stefano Lazzari, Paola Palermo, Maria Michela Palumbo, Federica Romitti, Irene Steinberg, Francesca Collino, Francesco Vassalli, Thomas Langer, Onnen Moerer, Leif Saager, Peter Herrmann, Paolo Cadringher, Konrad Meissner, Michael Quintel, Luciano Gattinoni

**Affiliations:** ^1^Department of Anesthesiology, University Medical Centre Göttingen, Göttingen, Germany; ^2^Department of Anesthesia, Intensive Care and Emergency, “Città della Salute e della Scienza” Hospital, Turin, Italy; ^3^Department of Medicine and Surgery, University of Milano-Bicocca, Milan, Italy; ^4^Department of Anesthesia and Intensive Care Medicine, Niguarda Ca’ Granda, Milan, Italy; ^5^Department of Anesthesiology, Intensive Care and Emergency Medicine Donau-Isar-Klinikum Deggendorf, Deggendorf, Germany

**Keywords:** ventilation-induced lung injury, mechanical ventilation, fluid balance, sodium retention, non-osmotic sodium

## Abstract

**Background:** Ventilator-induced lung injury (VILI) via respiratory mechanics is deeply interwoven with hemodynamic, kidney and fluid/electrolyte changes. We aimed to assess the role of positive fluid balance in the framework of ventilation-induced lung injury.

**Methods:***Post-hoc* analysis of seventy-eight pigs invasively ventilated for 48 h with mechanical power ranging from 18 to 137 J/min and divided into two groups: high vs. low pleural pressure (10.0 ± 2.8 vs. 4.4 ± 1.5 cmH_2_O; *p* < 0.01). Respiratory mechanics, hemodynamics, fluid, sodium and osmotic balances, were assessed at 0, 6, 12, 24, 48 h. Sodium distribution between intracellular, extracellular and non-osmotic sodium storage compartments was estimated assuming osmotic equilibrium. Lung weight, wet-to-dry ratios of lung, kidney, liver, bowel and muscle were measured at the end of the experiment.

**Results:** High pleural pressure group had significant higher cardiac output (2.96 ± 0.92 vs. 3.41 ± 1.68 L/min; *p* < 0.01), use of norepinephrine/epinephrine (1.76 ± 3.31 vs. 5.79 ± 9.69 mcg/kg; *p* < 0.01) and total fluid infusions (3.06 ± 2.32 vs. 4.04 ± 3.04 L; *p* < 0.01). This hemodynamic status was associated with significantly increased sodium and fluid retention (at 48 h, respectively, 601.3 ± 334.7 vs. 1073.2 ± 525.9 mmol, *p* < 0.01; and 2.99 ± 2.54 vs. 6.66 ± 3.87 L, *p* < 0.01). Ten percent of the infused sodium was stored in an osmotically inactive compartment. Increasing fluid and sodium retention was positively associated with lung-weight (*R*^2^ = 0.43, *p* < 0.01; *R*^2^ = 0.48, *p* < 0.01) and with wet-to-dry ratio of the lungs (*R*^2^ = 0.14, *p* < 0.01; *R*^2^ = 0.18, *p* < 0.01) and kidneys (*R*^2^ = 0.11, *p* = 0.02; *R*^2^ = 0.12, *p* = 0.01).

**Conclusion:** Increased mechanical power and pleural pressures dictated an increase in hemodynamic support resulting in proportionally increased sodium and fluid retention and pulmonary edema.

## Introduction

It is well known that positive pressure mechanical ventilation is associated with hemodynamic impairment and sodium and water retention (Drury et al., 1947). In the late 70 s, Hemmer and Suter suggested the use of vasoactive drugs rather than fluid infusions to achieve adequate perfusion while preventing fluid overload (Hemmer and Suter, 1979). In daily clinical practice, both fluids and cardiovascular drugs are commonly used to compensate for the detrimental hemodynamic effects of mechanical ventilation, despite the common notion that positive fluid balance is associated with worse outcomes (Mendes et al., 2020). However, it is not clear to what degree this is a simple association or a cause-effect relationship.

In a series of animal experiments aimed to elucidate some of the mechanisms of ventilator-induced lung injury (VILI), the animals often required large amounts of fluids and catecholamines to prevent hemodynamic collapse (Collino et al., 2019; Vassalli et al., 2020). This approach is similar to what it is routinely done in clinical practice: to maintain adequate hemodynamics the fluid infused into the patient can amount to several liters (Boyd et al., 2011). This is generally (and unfortunately) assumed to be an aesthetic rather than a harmful phenomenon, and at least not the primary cause of clinical worries (Vignon et al., 2020).

We hypothesized that an increased fluid balance may worsen the pulmonary function and VILI development, hence, the primary objective of the present study was to investigate whether the applied pleural pressure and positive fluid balance have a synergistic role in dictating the VILI severity, quantified by the increase of lung weight.

## Materials and Methods

In the present *post-hoc* analysis, data of seventy-eight pigs from two previous experimental studies investigating the relationship between mechanical power and VILI (Collino et al., 2019; Vassalli et al., 2020) were analyzed (see Supplementary Material). In the first study 36 pigs [weight (mean ± SD): 23.3 ± 2.3 kg] were ventilated for 48 h with tidal volume equal to the functional residual capacity, respiratory rate 30 bpm and different levels of PEEP: 0, 4, 7, 11, 14, and 18 cmH_2_O. The applied mechanical power ranged between 18 to 120 J/min (Collino et al., 2019).

In the second study, 42 pigs (mean weight 24.2 ± 2.0 kg) were ventilated for 48 h and were randomized into six groups, 3 of which at low (15 J/min) and 3 at high mechanical power (30 J/min). In each group, the targeted mechanical power was reached with different combinations of PEEP, tidal volume and respiratory rate. In the whole cohort, the tidal volume ranged from 0.5 to 3.8 L, the respiratory rate from 5 to 44 bpm and the PEEP from 5 to 25 cmH_2_O (Vassalli et al., 2020).

### Management of Experimental Animals

In both experiments, anesthesia was induced and maintained with propofol, midazolam and sufentanil. After intubation, volume-control mechanical ventilation was initiated with tidal volume 6 mL/kg, PEEP 5 cmH_2_O and a respiratory rate to maintain PaCO_2_ between 35 and 45 mmHg.

The animals had the following devices: endotracheal tube (size 7/7.5 mm); urinary catheter (5 Fr); adult esophageal catheter (8 Fr) Smartcath with esophageal balloon (the correct positioning of the esophageal catheter was checked by an end-expiratory occlusion test); central venous catheter (5 Fr) in the jugular vein, ultrasound-guided; swan-Ganz catheter (5 Fr) through an introducer (7 Fr) in the jugular vein, ultrasound guided; arterial PiCCO catheter (5 Fr) in the femoral artery, ultrasound guided. The target temperature was maintained throughout the experiment between 38 and 39°C (normal central temperature of pigs), by using a thermic blanket. Glycaemia was measured throughout the experiment, if values lower than 60 mg/dL were observed, dextrose 40% was initiated and maintained at the lowest infusion rate, in order to ensure glycemic levels between 60 and 100 mg/dL.

At the end of the experiment the animal was euthanized, autopsy was performed and lung, kidney, liver, muscle and bowel samples were sent to the pathology department. Six lung samples were collected for each lung (basal-ventral, basal-dorsal, central-ventral, central dorsal, apical-ventral, apical-dorsal) and one sample for the other organs. Wet-to-dry ratio was calculated as follow: each sample (~2 g of weight) was weighted before and after being heated and dried in an oven at 50 degrees, during 24 h.

Both studies were approved by the local ethic committee (nr. 16/2223 and nr. 18/2795, Niedersächsisches Landesamt für Verbraucherschutz und Lebensmittelsicherheit LAVES, Oldenburg, Niedersachsen, Germany) (Collino et al., 2019; Vassalli et al., 2020).

### Animal Experiments and Study Groups

For the purpose of this study, the animals were divided in two groups according to the median value of the calculated pleural pressure (Ppl_*mean*_) (Gattinoni et al., 2003):


$$P_{p\,l_{m\,e\,a\,n}=}\,P_{a\,w_{m\,e\,a\,n}}\cdot \frac{E_{C\,W}}{E_{R\,S}}$$

where Paw_*mean*_ is the mean airway pressure, E_*CW*_ is the chest-wall elastance, and E_*RS*_ is the total elastance of the respiratory system. Pleural pressure was chosen as the study variable because it is the most established determinant of hemodynamic changes, in mechanical ventilation.

### Measured Variables

*Respiratory mechanics*: plateau pressure, PEEP, tidal volume and esophageal pressure were recorded hourly, as well as their derived variables: total/chest-wall/lung elastances and total/lung mechanical power.

*Hemodynamic variables*: heart rate, pulmonary/systemic blood pressure, cardiac output, pulmonary/systemic vascular resistances were measured every 6 h.

*Laboratory variables:* sodium concentration (in plasma and urine) and osmolarity (in plasma) were measured at 0, 6, 12, 24, and 48 h.

*Sodium retention* (Na^+^_*ret*_; mmol) was computed at each timepoint as the infused sodium minus the sodium excreted by urine (see Supplementary Material for detailed explanation and equation).

*Fluid balance* (V_*ret*_) was computed as the amount of infused fluids minus the urinary output.

### Fluid Management

Maintenance fluid consisted of 1–2 mL/h of Sterofundin infusion throughout the experiment. Additional fluids were administered in form of Sterofundin or Gelafundin ISO 4% fluid challenge (250 mL in 5–10 min) if: MAP < 60 mmHg; clinical signs of hypoperfusion (raised lactates, skin mottling, decreased urine output); hemodynamic monitoring dynamic indices (pulse pressure variation > 13% or systolic volume variation > 12%). Norepinephrine was initiate if the animal was not fluid responsive.

### Calculation of Sodium Infusion

Milliliters of infused solutions times the sodium content of normal saline (0.154 mmol/mL), Sterofundin (0.145 mmol/mL) and Gelafundin ISO 4% (0.151 mmol/mL). All fluids were included in the analysis either if they were infused as fluid challenge, maintenance or for drug dilution.

### Calculation of Retained Sodium and Fluids

*Retained sodium*: amount of infused fluid times the sodium concentration of the specific fluid (normal saline 154 mmol/L, sterofundin 145 mmol/L and gelafundin ISO 4% 151 mmol/L), minus the amount of urine in milliliters times the urinary sodium concentration:


Na=+ret[Na]+×infV-inf[Na]+×uVu

where [Na^+^]_*inf*_ (mmol/L) is the sodium concentration of the infused fluids, V_*inf*_ (L) is the amount of fluid infused during an experiment interval, [Na^+^]_*u*_ (mmol/L) and V_*u*_ (L) are the urine sodium concentration and volume during the same interval. We only included the fraction of dissociated sodium, and excluded the sodium bound to drug molecules.

*Fluid balance* (V_*ret*_) was computed as the amount of infused fluids minus the urinary output:


V=retV-infVu

where V_*inf*_ (L) is the infused fluid and V_*u*_ (L) is the urinary output.

### Two-Compartment Kinetic Model and Mass Balance Equations

To investigate the sodium distribution between intracellular/extracellular compartments and the non-osmotic sodium storage, the following assumptions were made: (1) extracellular and intracellular compartments equal 20 and 40% of the pig body weight (Svensson et al., 2020); (2) the osmotic concentration between the two compartments is equal and the equilibrium is maintained by means of water shifts; (3) the count of milliosmoles in the extracellular fluid at the end of any timepoint must be equal to the initial amount of milliosmoles plus the milliosmoles due to the retained sodium (Na^+^_*ret*_ × 2); (4) the milliosmoles exceeding this equilibrium were classified as missing sodium (Na^+^_*miss*_), i.e., sodium stored in a non-osmotic form.

**FIGURE 1:**
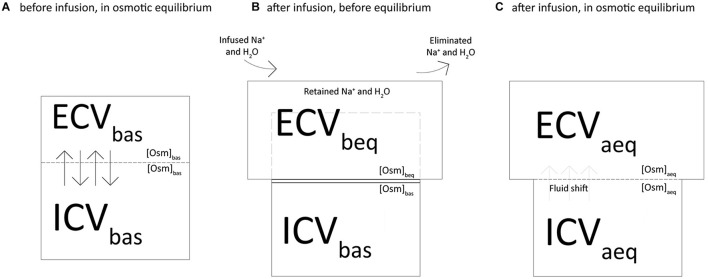
Two-chamber kinetic model to assess the distribution of sodium and fluids across the body compartments.

Figure 1 depicts the two-chamber kinetic model we applied to assess the distribution of sodium and fluids across the body compartments. At baseline, the extracellular (ECV_*bas*_) and intracellular (ICV_*bas*_) volumes were assumed to be in osmotic equilibrium (Osm_*bas*_), i.e., the osmolar concentration (Osm_*bas*_) was equal in ECV_*bas*_ and ICV_*bas*_ (Figure 1A). As shown in Figure 1B, the net retained sodium and fluid (infused minus excreted) are confined within the extracellular volume, i.e., before the osmotic equilibrium (ECV_*beq*_). In this condition, the osmotic and sodium concentrations are higher in the extracellular volume and therefore, to reach the osmotic equilibrium, water must shift from the intracellular to the extracellular compartment. Furthermore (Figure 1C), we assumed the osmotic concentration to be equal between compartments (ECV_*aeq*_ and ICV_*aeq*_) and the osmotic equilibrium is achieved through the fluid shift from compartments.

The following equations were used to calculate osmolarity, extracellular and intracellular volumes, fluid shift and sodium concentration:

(1) We computed the osmolarity (Osm_*beq*_) and the extracellular volume (ECV_*beq*_) before the osmotic equilibrium as shown in Figure 1B, according to the following equations:


[Osm]=beq([Osm]×basECV+bas2Na)+ret/(ECV+basV)retECV=beqECV+basVret

Where [Osm]_*bas*_ is osmolarity at baseline, ECV_*bas*_ is the baseline extracellular volume, Na^+^_*ret*_ is the net retained sodium and V_*ret*_ is the fluid retained.

(2) We computed the osmolarity at equilibrium ([Osm]_*aeq*_) and the fluid required to reach it (V_*shift*_) with the following equations (Figure 1C):


[Osm]=aeq([Osm]×beqECV+beq[Osm]×basICV)bas/(ECV+basICV+basV)ret

V=shift([Osm]×beqECV-beq[Osm]×aeqECV)beq/[Osm]aeq

Where ICV_*bas*_ is the baseline intracellular volume.

(3) We computed the new values of extracellular (ECV_*aeq*_) and intracellular (ICV_*aeq*_) volumes after the osmotic equilibrium (Figure 1C):


ECV=aeqECV+beqVshift

ICV=aeqICV-basVshift

(4) The following modified mass balance equations were used to calculate the expected sodium concentrations in plasma before ([Na^+^]_*beq*_) and after ([Na^+^]_*aeq*_) the osmotic equilibrium:


[Na]+=beq([Na]+×basECV+basNa)+ret/(ECV+basV)ret

[Na]+=aeq([Na]+×basECV+basNa)+ret/(ECV+basV+retV)shift

where [Na^+^]_*bas*_ is sodium plasma concentration at baseline.

(5) Finally, we used the following equation to assess the amount of sodium that justifies the difference between the measured and the calculated sodium concentration after osmotic equilibrium (Na^+^_*miss*_):


Na=+miss[Na]+×basECV+basNa-+ret[Na]+act×(ECV+basV+retV)shift

where [Na^+^]_*act*_ is the actually measured concentration of sodium at a specific experimental time.

### Statistical Analysis

Data are reported as mean ± standard deviation. Baseline and end-experiment differences between groups were assessed by Student’s *t*-test. The strength of the relationship between variables was tested with linear regression. We evaluated the effect of time and accounted for the repeated measures design by using a linear mixed effects model, where the fixed effect was the pleural pressure group and the random effect the animal ID. Two-tailed *p*-values < 0.05 were considered statistically significant. All analyses were performed with R for Statistical Computing 4.0.2.

## Results

In Table 1 we reported the respiratory mechanics measured in both groups: all mechanical variables, except for respiratory rate and lung elastance, differed between the groups throughout the experiment. The difference in pleural pressure was maintained nearly unaltered throughout the experiment (see Supplementary Figure 1). The amount of fluids and electrolytes infused in the two groups is summarized in Supplementary Table 1.

**TABLE 1:** Respiratory mechanics and hemodynamics in high and low pleural pressure groups.

Variables	Values at 0.5 h			Means (averaged over the four time-points: 6, 12, 24, 48 h)		
		
	Low P_*pl*_ group	High P_*pl*_ group	*p*-value	Low P_*pl*_ group	High P_*pl*_ group	*p*-value
Pleural pressure [cmH_2_O]	4.6 (1.5)	9.4 (2.0)	<0.01	4.4 (1.5)	10.0 (2.8)	<0.01
Plateau pressure [cmH_2_O]	25.4 (12.6)	35.4 (11.9)	<0.01	24.5 (8.3)	35.5 (10.4)	<0.01
PEEP [cmH_2_O]	7.9 (6.6)	15.3 (6.5)	<0.01	6.9 (5.1)	16.8 (6.6)	<0.01
Respiratory rate [bpm]	26 (11)	26 (10)	0.78	27 (11)	26 (10)	0.30
Tidal volume [mL]	438 (235)	391 (149)	0.31	447 (227)	375 (133)	<0.01
Total elastance [cmH_2_0/L]	42.7 (19.0)	54.9 (22.5)	0.01	42.3 (12.6)	52.1 (17.0)	<0.01
Lung elastance [cmH_2_0/L]	28.0 (19.2)	32.6 (20.0)	0.31	27.7 (12.1)	30.6 (14.1)	0.06
Mechanical power [J/min]	45.5 (26.1)	67.8 (27.3)	<0.01	42.9 (16.5)	70.8 (26.1)	<0.01
Lung mechanical power [J/min]	31.7 (26.2)	39.7 (22.8)	0.15	28.3 (14.7)	41.3 (19.5)	<0.01
Heart rate [bpm]	112 (29)	135 (24)	<0.01	97 (22)	113 (27)	<0.01
Systolic arterial pressure [mmHg]	100.1 (13.0)	103.0 (15.1)	0.38	104.7 (16.2)	103.3 (15.5)	0.43
Diastolic arterial pressure [mmHg]	60.1 (10.4)	64.2 (13.6)	0.14	57.0 (13.4)	55.6 (12.2)	0.37
Mean arterial pressure [mmHg]	75.7 (10.8)	80.2 (12.6)	0.10	73.5 (12.5)	73.0 (12.8)	0.73
Central venous pressure [mmHg]	8.9 (3.5)	12.0 (4.8)	<0.01	8.5 (4.1)	13.2 (5.1)	<0.01
Systolic pulmonary pressure [mmHg]	28.2 (8.3)	34.6 (8.6)	<0.01	26.8 (7.1)	36.3 (10.0)	<0.01
Diastolic pulmonary pressure [mmHg]	17.6 (6.9)	23.0 (6.7)	<0.01	16.0 (5.3)	23.2 (7.1)	<0.01
Mean pulmonary pressure [mmHg]	22.6 (7.3)	29.0 (7.0)	<0.01	21.4 (6.2)	29.7 (8.2)	<0.01
Wedge pressure [mmHg]	13.1 (5.8)	17.8 (7.3)	<0.01	11.8 (5.5)	17.4 (5.8)	<0.01
Cardiac output [L/min]	3.79 (1.03)	4.02 (1.24)	0.40	2.96 (0.92)	3.41 (1.68)	<0.01
Systemic vascular resistances [(dyn × s)/L]	1,588(427)	1,526(671)	0.64	1,924(601)	1,600(672)	<0.01
Pulmonary vascular resistances [(dyn × s) /L]	218 (122)	237 (112)	0.51	267 (126)	319 (170)	<0.01
Infused fluids [L]	0.41 (0.27)	0.73 (0.44)	<0.01	3.06 (2.32)	4.04 (3.04)	<0.01
Infused dose of norepinephrine [mcg/kg]	0.01 (0.02)	0.07 (0.09)	<0.01	1.76 (3.31)	5.79 (9.69)	<0.01

*Data expressed as: mean (SD). p-value assessed with Student’s t-test. Number of animals at 0.5 h: 78. Number of animals at 6 h: 78; number of animals at 48 h: 65.*

### Sodium Retention and Pleural Pressures

**FIGURE 2:**
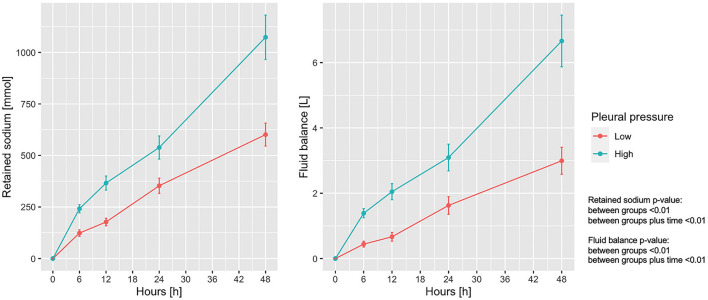
Trend of retained sodium and fluid balance according to pleural pressure. Line-plot over the 48 h of the experiment. Statistic: linear mixed effects model. Retained sodium: data available in 78 animals at 0 h and in 60 at 48 h (36 in low-pressure group and 24 in the high-pressure group). Fluid balance: data available in 78 animals at 0 h and in 62 at 48 h (38 in low-pressure group and 24 in the high-pressure group).

Sodium retention and fluid balance are presented in Figure 2 as a function of the experimental time. As shown, sodium retention differed significantly between the groups and increased significantly increased over time. The mean ± SD end-experiment sodium retention was 601.3 ± 334.7 and 1,073.2 ± 525.9 mmol in the low and high pleural pressure groups, respectively (*p* < 0.01). Similarly, the fluid balance increased significantly over time, and was significantly larger in the high pleural pressure group (at 48 h: 2.99 ± 2.54 vs. 6.66 ± 3.87 L; *p* < 0.01). The fluid balance in both groups was similar to the difference in weight-gain measured at the end of the experiment: 2.8 ± 1.8 vs. 5.5 ± 3.0 Kg; (*p* < 0.01). The positive fluid balance derived by a combination of greater amount of infused fluids and a decrease in urinary output: at the end of the experiment 5.72 ± 2.40 vs. 8.76 ± 3.40 L (*p* < 0.01) and 2.72 ± 0.89 vs. 2.10 ± 0.93 L (*p* = 0.01), respectively (Supplementary Figure 2).

### Sodium Retention and Hemodynamics

**FIGURE 3:**
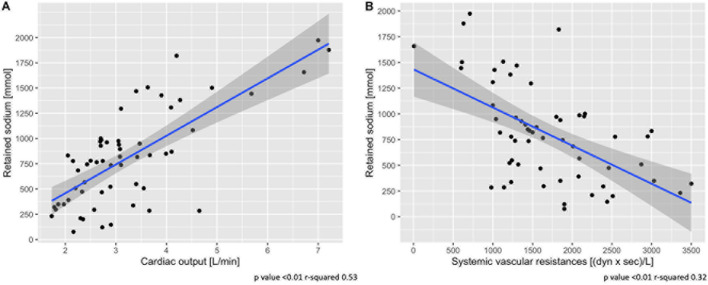
Association between sodium retention and cardiac output **(A)** and systemic vascular resistances **(B)**. Scatter-plot of measures obtained at 48 h. Statistic: linear logistic regression. Data available in 58 animals for cardiac output and vascular resistances.

Table 1 shows the hemodynamic data from the two groups: heart rate, central venous pressure and pulmonary pressures were higher in the high pleural pressure group (all *p* < 0.01). Cardiac output was higher in the high pleural pressure group, and unexpectedly, it was significantly associated with greater sodium retention (Figure 3A). The systemic vascular resistances were higher in the low pleural pressure group and associated with less sodium retention, contrary to what was expected (Figure 3B). It must be noted that cardiac output and vascular resistances were a function of the infused fluids (Supplementary Figure 3) and the dose of catecholamines (Supplementary Figure 4).

### Sodium Distribution

**FIGURE 4:**
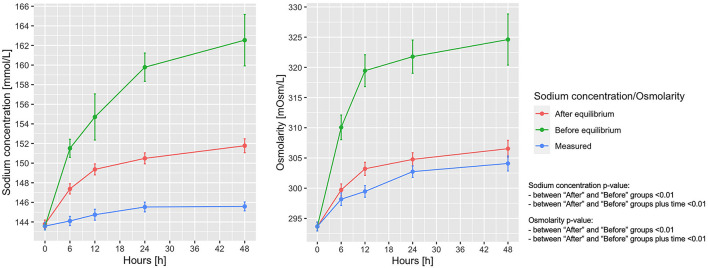
Measured and calculated sodium concentration and osmolarity before and after osmotic equilibrium. Line-plot over the 48 h of the experiment. Statistic: linear mixed effects model comparing the “before” and “after” osmotic equilibrium values. Sodium concentration: data available in 78 animals at 0 h for the three items, and in 65 at 48 h for measured sodium, 65 for expected sodium before equilibrium, and 55 for expected sodium after equilibrium. Osmotic concentration: data available in 78 animals at 0 h in the three items, and in 65 for measured osmotic concentration, 55 for expected osmotic concentration before equilibrium, and 55 for expected osmotic concentration after equilibrium.

In Figure 4A shows the measured vs. the expected sodium concentration in the entire animal cohort, if all sodium and fluids had remained within the extracellular space (before the osmotic equilibrium), compared with the values obtained after the osmotic equilibrium with the intracellular space. As shown, the sodium concentration at 48 h calculated before and after osmotic equilibrium would be 162.5 ± 21.2 and 151.8 ± 5.2 mmol/L, respectively, instead of the measured 145.6 ± 3.7 mmol/L. Osmolarity before and after osmotic equilibrium would amount to 324.6 ± 31.4 and 306.5 ± 10.3 mOsm/L respectively, compared with the measured 304.1 ± 10.0 mOsm/L (Figure 4B). The calculated extracellular and intracellular volumes at 48 h were 10.04 ± 3.37 and 9.12 ± 0.84 L, after shifting 0.40 ± 0.33 L of water from the intracellular to the extracellular compartments (Supplementary Figure 5).

According to the calculated sodium concentrations before and after osmotic equilibrium, 74.9 ± 41.8 mmol of sodium out of 765.8 ± 479.2 mmol of the total retained sodium have to be removed from the extracellular volume to account for the measured sodium osmotic equilibrium.

### Contribution of Sodium Retention to Lungs’ Weight and Wet-to-Dry Ratio

At the end of the experiment, the weight of the lungs (463.2 ± 158.2 vs. 578.2 ± 162.6 g; *p* < 0.01) and the lung wet-to-dry ratio (6.38 ± 0.73 vs. 6.72 ± 0.58; *p* = 0.04) were significantly different between the low and high-pressure groups; Supplementary Figure 6 shows the positive association between pleural pressure, and lung-weight and wet-to-dry ratio.

**FIGURE 5:**
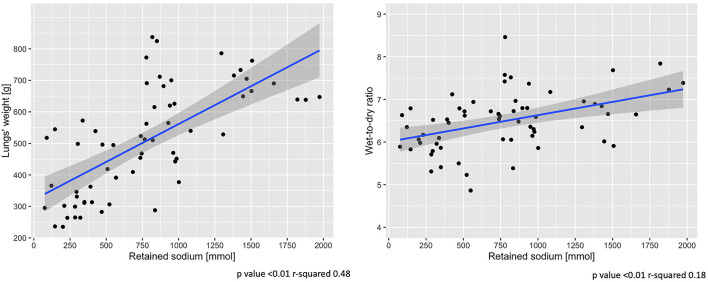
End-of-experiment association between lung-weight and wet-to-dry ratio vs. retained sodium. Scatter-plot of measures obtained at 48 h. Statistic: linear logistic regression. Data available in 62 animals for lungs’ weight and wet-to-dry ratio.

An increase of sodium and fluid retention was positively associated with a higher lung-weight (*R*^2^ = 0.48, *p* < 0.01; *R*^2^ = 0.43, *p* < 0.01) and higher wet-to-dry ratio of lung (*R*^2^ = 0.18, *p* < 0.01; *R*^2^ = 0.14, *p* < 0.01) and kidney (*R*^2^ = 0.12, *p* = 0.01; *R*^2^ = 0.11, *p* = 0.02). No association was found between sodium retention and the wet-to-dry ratio of liver, bowel and muscle (see Supplementary Table 2). As shown in Figure 5, at 48 h the increasing amount of retained sodium was significantly associated with an increase in lung-weight and wet-to dry-ratio.

## Discussion

The main findings of the present study are: (1) a higher pleural pressure leads to an increase in fluid and sodium retention; (2) sodium and fluid retention were greater when cardiac output was higher and the systemic vascular resistance was lower; (3) about 10% of the retained sodium must be osmotically inactive to account for the measured sodium and osmotic equilibrium, and (4) pulmonary edema and the wet-to-dry ratio were positively associated with the amount of retained sodium and fluid.

The effects of mechanical ventilation on the sodium and fluid balance have been known since the first use of positive pressure ventilation (Cournand and Motley, 1948). Mechanical ventilation with positive pressure is an easy way to increase the intrathoracic pressure, whose immediate effect is a decrease in cardiac output proportional to the increase in intrathoracic pressure. We do not know exactly, among tidal volume, mean airway pressure, driving pressure or PEEP which is the main responsible for fluid retention. Previous experiments strongly suggest, however, that the PEEP level may play the most relevant role (Marshall et al., 1982).

The mechanisms of sodium and water retention as a consequence of decreased cardiac output and cardiac failure were described decades ago (Pinsky et al., 1983). Other mechanisms have been described to cause sodium and fluid retention, as the amount of infused sodium and the chloride dependent glomerulotubular feedback (Wilcox, 1983; Hansen et al., 1998). In any case, however, these mechanisms cannot operate alone in spontaneously breathing non-anesthetized animals, which respond to the fluid load by a proportional increase of urinary output.

Our experiments were designed to study the effects of mechanical power on the lung parenchyma, collectively referred to as VILI. As the experiments were performed in healthy animals, the intensity of ventilation and the thoracic pressure required to produce lung damage were substantially elevated. Hemodynamics were severely compromised immediately after the increase in intrathoracic pressure, and, as a consequence, we observed a significant decrease in sodium excretion and urine volume contraction. This homeostatic response was maintained during the experiment as sodium retention and fluid balance increased linearly until the end of the experiment (Figure 2).

As discussed earlier, the decrease in cardiac output is the generally recognized mechanism that explains sodium retention by activating several hormonal pathways. Schrier (1988) proposed a unifying theory for volume regulation, where the arterial central volume is the regulating variable, through the baroreceptors, of the body fluid volume. Accordingly, an increase in intrathoracic pressure may lead to a decrease in cardiac output and in turn to a decrease in arterial intrathoracic volume. Surprisingly, however, we found that the cardiac output and sodium retention in our experiments were positively associated, i.e., the higher the cardiac output, the larger the amount of retained sodium. This is exactly the opposite of what we may expect from Schrier’s unifying theory, and in general, from what is observed in cardiac failure. We believe that the possible explanation of our observations may depend on two primary factors. First of all, in order to maintain the circulation according to the strict protocol, the study doctors were administering large volumes of fluids, primarily guided by the arterial pressure. And second, there was a large time lag between the circulatory support interventions, which were a continuous process, and when we obtained the hemodynamic and sodium balance data at the fixed 6-h intervals. Therefore, a possible explanation is that the animal reacted immediately to the abnormal increase in pleural pressure with a decrease in cardiac output, and possibly systolic arterial pressure, to which the immediate response was to infuse fluids and cardiovascular drugs according to the study protocol. This phenomenon is depicted in Supplementary Figure 7, where a constant systolic arterial pressure is maintained over a wide range of fluid or norepinephrine infusion rates. Despite the large infused volume, it is likely that only a fraction remained in the vascular compartment to adequately correct the central arterial volume. Thus, the decrease in the central arterial volume maintained the hormonal and kidney pathways that were activated to enhance sodium and water retention.

Although several studies have investigated the theory and practice of sodium distribution (Titze et al., 2014), the literature related to the distribution of the infused sodium is surprisingly scanty. Actually, the technology required to assess the destiny of the infused sodium require measurements of body compartments as well as the tracing the electrolytes of interest. In our experiment we did not measure body compartments nor did we trace the infused sodium. However, we attempted to determine whether a general understanding on sodium distribution could be obtained by utilizing the measured osmolarity and sodium concentrations. Over the past decades, several formulas have been proposed to estimate changes in plasma sodium after infusing sodium solutions (Fazekas et al., 2013). These are primarily based on the concept of exchangeable sodium and potassium and assume that all the sodium ultimately retained after urinary elimination is confined within the extracellular compartment. This concept, however, was challenged in recent years by a precise study of the sodium balance in long term human experiments. It was proposed that part of the sodium is retained in compartments where it has no osmotic action (Olde Engberink et al., 2017), e.g., in the glycosaminoglycan of bone, cartilage, and skin (Titze, 2009; Hofmeister et al., 2015; Olde Engberink et al., 2015).

In our experiment we were able to separately analyze the distribution of sodium and osmotically active solute particles between the extracellular and intracellular compartments. With this approach, at the end of the experiment, we observed the following: (a) an increase in the extracellular compartment of 111.4% (from 4.75 to 10.04 L) due to the retained volume after the infusions (92.4%) and to the volume shift from the intracellular fluid (7.6%; 0.40 L) (Supplementary Figure 5), (b) a 3.4% decrease in intracellular volume (9.50–9.18 L) (Supplementary Figure 5), and (c) 9.8% (74.0 mmol) of the retained sodium was not justified by the measured sodium and osmotic concentrations. This could be due to possible inaccuracies in our mass balance calculations or, alternatively, to accumulation of sodium in the non-osmotic storage compartments recently described (Olde Engberink et al., 2017). However, a similar percentage of “missing sodium” was computed by analyzing previous experiments performed in a different center by different personnel with a different experimental setup (Langer et al., 2012). Also in this case, the possible non-osmotic storage accounted for the 13.6% of the infused sodium (see Supplementary Material for the analysis).

The most striking results, however, are the clinical consequences of sodium and fluid retention in the framework of VILI. Actually, the present work was based on two large experiments aimed at investigating whether the level of mechanical power induced a proportional level of lung injury (Collino et al., 2019; Vassalli et al., 2020). Several data were acquired in both experiments: the lung-weight and wet-to-dry ratio showed a trend toward a greater increase in pigs with higher mechanical power, without achieving statistical significance in the single experiments. A significant association between lung-weight and mechanical power was reached only analyzing the data of the experiments together. Considering the data as a whole, it is likely that what we call “VILI” should be intended as a combination of structural changes of the lung, inflammatory reaction and pulmonary edema, in recognition of the hemodynamic effects on the lung structure and function. The latter, regardless the structural changes, may be exacerbated by the need for large fluid volumes to be infused in order to maintain the circulation during high pressure/volume ventilation.

## Conclusion

In conclusion, increasing the intrathoracic pressure led to a significant increase in sodium and fluid retention, which may play a significant role in VILI induced by excessive mechanical power.

## Data Availability Statement

The raw data supporting the conclusions of this article will be made available by the authors, without undue reservation.

## Ethics Statement

The animal study was reviewed and approved by the Niedersächsisches Landesamt für Verbraucherschutz und Lebensmittelsicherheit (LAVES). Oldenburg (Niedersachsen), Germany. Protocol nrs. 16/2223 and 18/2795.

## Author Contributions

SG and LG: conception and design. IP, MB, FR, FC, and FV: data acquisition. SG, MB, SL, PP, MP, FR, IS, PH, TL, and PC: analysis and interpretation. OM, LS, KM, MQ, and LG: drafting the manuscript for important intellectual content. All authors contributed to the article and approved the submitted version.

## Conflict of Interest

LG reports a consultancy for General Electrics and SIDAM. He also receives lectures fees from Estor and Dimar. LS reports financial relationships with Medtronic, Ferrer Deutschland and Merck. Part of the salary support for the author MB was provided by an unrestricted research grant from Sartorius Inc. Göttingen, Germany. The remaining authors declare that the research was conducted in the absence of any commercial or financial relationships that could be construed as a potential conflict of interest.

## Publisher’s Note

All claims expressed in this article are solely those of the authors and do not necessarily represent those of their affiliated organizations, or those of the publisher, the editors and the reviewers. Any product that may be evaluated in this article, or claim that may be made by its manufacturer, is not guaranteed or endorsed by the publisher.

## References

[B1] Boyd J. H. Forbes J. Nakada T. A. Walley K. R. Russell J. A. (2011). Fluid resuscitation in septic shock: a positive fluid balance and elevated central venous pressure are associated with increased mortality. *Crit. Care Med.* 39 259–265. 10.1097/ccm.0b013e3181feeb15 20975548

[B2] Collino F. Rapetti F. Vasques F. Maiolo G. Tonetti T. Romitti F. (2019). Positive end-expiratory pressure and mechanical power. *Anesthesiology* 130 119–130. 30277932 10.1097/ALN.0000000000002458

[B3] Cournand A. Motley H. L. (1948). Physiological studies of the effects of intermittent positive pressure breathing on cardiac output in man. *Am. J. Physiol.* 152 162–174. 10.1152/ajplegacy.1947.152.1.162 18903440

[B4] Drury D. R. Henry J. P. Goodman J. (1947). The effects of continuous pressure breathing on kidney function. *J. Clin. Invest.* 26 945–951. 10.1172/jci101889 16695498 PMC439393

[B5] Fazekas A. S. Funk G. C. Klobassa D. S. Rüther H. Ziegler I. Zander R. (2013). Evaluation of 36 formulas for calculating plasma osmolality. *Intensive Care Med.* 39 302–308. 10.1007/s00134-012-2691-0 23081685

[B6] Gattinoni L. Carlesso E. Cadringher P. Valenza F. Vagginelli F. Chiumello D. (2003). Physical and biological triggers of ventilator-induced lung injury and its prevention. *Eur. J. Respir. Suppl.* 47 15s–25s. 10.1183/09031936.03.00021303 14621113

[B7] Hansen P. B. Jensen B. L. Skott O. (1998). Chloride regulates afferent arteriolar contraction in response to depolarization. *Hypertension* 32 1066–1070. 10.1161/01.hyp.32.6.1066 9856975

[B8] Hemmer M. Suter P. M. (1979). Treatment of cardiac and renal effects of PEEP with dopamine in patients with acute respiratory failure. *Anesthesiology* 50 399–403. 10.1097/00000542-197905000-00005 378029

[B9] Hofmeister L. H. Perisic S. Titze J. (2015). Tissue sodium storage: evidence for kidney-like extrarenal countercurrent systems? *Pflugers Arch.* 467 551–558. 10.1007/s00424-014-1685-x 25600900 PMC4340694

[B10] Langer T. Carlesso E. Protti A. Monti M. Comini B. Zani L. (2012). In vivo conditioning of acid-base equilibrium by crystalloid solutions: an experimental study on pigs. *Intensive Care Med.* 38 686–693. 10.1007/s00134-011-2455-2 22273748

[B11] Marshall B. E. Berry A. J. Marshall C. Geer R. T. (1982). Influence of ventilation on response to fluid load in dogs: body water and albumin distribution. *Anesthesiology* 57 103–110. 10.1097/00000542-198208000-00007 7046518

[B12] Mendes R. D. S. Pelosi P. Schultz M. J. Rocco P. R. M. Silva P. L. (2020). Fluids in ARDS: more pros than cons. *Intensive Care Med. Exp.* 8(Suppl. 1):32. 10.1186/s40635-020-00319-x PMC7746428 33336259

[B13] Olde Engberink R. H. G. Rorije N. M. G. Homan van der Heide J. J. van den Born B. J. Vogt L. (2015). Role of the vascular wall in sodium homeostasis and salt sensitivity. *J. Am. Soc. Nephrol.* 26 777–783. 10.1681/asn.2014050430 25294232 PMC4378110

[B14] Olde Engberink R. H. G. Rorije N. M. G. van den Born B. H. Vogt L. (2017). Quantification of nonosmotic sodium storage capacity following acute hypertonic saline infusion in healthy individuals. *Kidney Int.* 91 738–745. 10.1016/j.kint.2016.12.004 28132715

[B15] Pinsky M. R. Summer W. R. Wise R. A. Permutt S. Bromberger-Barnea B. (1983). Augmentation of cardiac function by elevation of intrathoracic pressure. *J. Appl. Physiol. Respir. Environ. Exerc. Physiol.* 54 950–955. 10.1152/jappl.1983.54.4.950 6853301

[B16] Schrier R. W. (1988). Pathogenesis of sodium and water retention in high-output and low-output cardiac failure, nephrotic syndrome, cirrhosis, and pregnancy (1). *N. Eng. J. Med.* 319 1065–1072. 10.1056/nejm198810203191606 3050518

[B17] Svensson R. Zdolsek J. Malm M. Hahn R. G. (2020). Electrolyte-based calculation of fluid shifts after infusing 0.9% saline in severe hyperglycemia. *Intensive Care Med. Exp.* 8:59. 10.1186/s40635-020-00345-9 PMC7554273 33048297

[B18] Titze J. (2009). Water-free sodium accumulation. *Semin. Dial.* 22 253–255. 10.1111/j.1525-139x.2009.00569.x 19573004

[B19] Titze J. Dahlmann A. Lerchl K. Kopp C. Rakova N. Schröder A. (2014). Spooky sodium balance. *Kidney Int.* 85 759–767. 10.1038/ki.2013.367 24107854

[B20] Vassalli F. Pasticci I. Romitti F. Duscio E. Aßmann D. J. Grünhagen H. (2020). Does iso-mechanical power lead to iso-lung damage? An experimental study in a porcine model. *Anesthesiology* 132 1126–1137. 10.1097/aln.0000000000003189 32032095

[B21] Vignon P. Evrard B. Asfar P. Busana M. Calfee C. S. Coppola S. (2020). Fluid administration and monitoring in ARDS: which management? *Intensive Care Med.* 46 2252–2264. 10.1007/s00134-020-06310-0 33169217 PMC7652045

[B22] Wilcox C. S. (1983). Regulation of renal blood flow by plasma chloride. *J. Clin. Invest.* 71 726–735. 10.1172/jci110820 6826732 PMC436923

