# Supplementary material for: Role of Fluid and Sodium Retention in Experimental Ventilator-Induced Lung Injury
Source: Front Physiol. 2021 Sep 13;12:743153. doi: 10.3389/fphys.2021.743153 (PMC8473803; doi:10.3389/fphys.2021.743153)
Supplement: Supplementary file 1 [file Data_Sheet_1.docx]

**Role of fluid and sodium retention in experimental ventilator-induced lung injury**

**Supplementary material**

Simone Gattarello*^1^, Iacopo Pasticci^1^, Mattia Busana^1^, Stefano Lazzari^1^, Paola Palermo^1^, Maria Michela Palumbo^1^, Federica Romitti^1^, Irene Steinberg^1^, Francesca Collino^1^, Francesco Vassalli^1^, Thomas Langer^2^, Onnen Moerer^1^, Leif Saager^1^, Konrad Meissner^1^, Peter Herrmann^1^, Paolo Cadringher^1^, Michael Quintel^1,3^ and Luciano Gattinoni^1^.

**Affiliations:**

1. Department of Anesthesiology, University Medical Centre Göttingen, Göttingen, Germany.
2. Department of Anesthesia, Intensive Care and Emergency, "Città della Salute e della Scienza" Hospital, Turin, Italy.
3. Department of Medicine and Surgery, University of Milano-Bicocca. Department of Anesthesia and Intensive Care Medicine, Niguarda Ca’ Granda, Milan, Italy.
4. Department of Anesthesiology, Intensive Care and Emergency Medicine Donau-Isar-Klinikum Deggendorf, Deggendorf, Germany.

**Corresponding author:**

Simone Gattarello, MD, PhD

Department of Anesthesiology, University Medical Center Göttingen

Robert Koch Straße 40, 37075, Göttingen, Germany

gattarello@gmail.com

**Supplemental methods:**

*Calculated respiratory variables:*

The following variables were calculated/computed (Vt: tidal volume, RR: respiratory rate, E: elastance, MP: mechanical power):

- Mean pleural pressure (cmH_2_O) = Paw_mean_ x (E_chest-wall_‎ / E_respiratory-system_)

- Respiratory system elastance (cmH_2_O/L) = (Paw_plat_ – Paw_PEEP_) / Vt

- Chest wall elastance (cmH_2_O/L) = (Pes_plat_ – Pes_PEEP_) / Vt

- Lung elastance (cmH_2_O/L) = [(Paw_plat_ – Paw_PEEP_) – (Pes_plat_ – Pes_PEEP_)] / Vt

- Respiratory system mechanical power (J/min) = 0,098 x RR x Vt x [Paw_peak_ - (P_pl_ - PEEP) / 2]

- Lung mechanical power (J/min) = MP_respiratory-system_ x E_lung_  / E_respiratory-system_

Supplementary results:

Table S1: proportion of infused fluids, concentration of the infused sodium and chloride and total amount of infused and excreted sodium and chloride in low and high pleural pressure groups at 48h.

| **Variables** | **Low Pleural pressure** | **High Pleural pressure** | **p-value** |
| --- | --- | --- | --- |
| Sterofundin (%) | 0.59 (0.13) | 0.43 (0.11) | <0.01 |
| Normal saline (%) | 0.09 (0.08) | 0.10 (0.09) | 0.54 |
| Gelafundin (%) | 0.31 (0.13) | 0.45 (0.11) | <0.01 |
| [Na]_infused_ (mmol/L) | 147.7 (0.9) | 148.7 (0.8) | <0.01 |
| [Cl]_infused_ (mmol/L) | 122.1 (4.5) | 119.0 (4.3) | 0.01 |
| Na_infused_ (mmol) | 731.9 (343.5) | 1186.2 (499.3) | <0.01 |
| Na_eliminated_ (mmol) | 168.4 (101.7) | 113.0 (95.4) | 0.04 |
| Cl_infused_ (mmol) | 589.9 (269.) | 928.2 (376.3) | <0.01 |
| Cl_eliminated_ (mmol) | 171.5 (103.4) | 120.5 (109.3) | 0.07 |

Data expressed as: mean (SD)

Table S2: association between sodium retention and wet-to-dry ratio

| **Variables** | **Odds Ratio (95% CI)** | **p-value** |
| --- | --- | --- |
| Wet-to-dry lung | 0.001 (<0.001-0.001) | <0.01 |
| Wet-to-dry liver | <0.001 (<0.001-<0.001) | 0.68 |
| Wet-to-dry kidney | 0.001 (<0.001-0.001) | 0.01 |
| Wet-to-dry bowel | <0.001 (<0.001-0.001) | 0.38 |
| Wet-to-dry muscle | <0.001 (-0.001-<0.001) | 0.38 |

Figure S1: Trend of pleural pressure at baseline and throughout the experiment in the two groups





Figure S2: Infused fluids and urinary output, according to pleural pressure





Figure S3: Association between cardiac output and vascular resistances vs. infused fluids


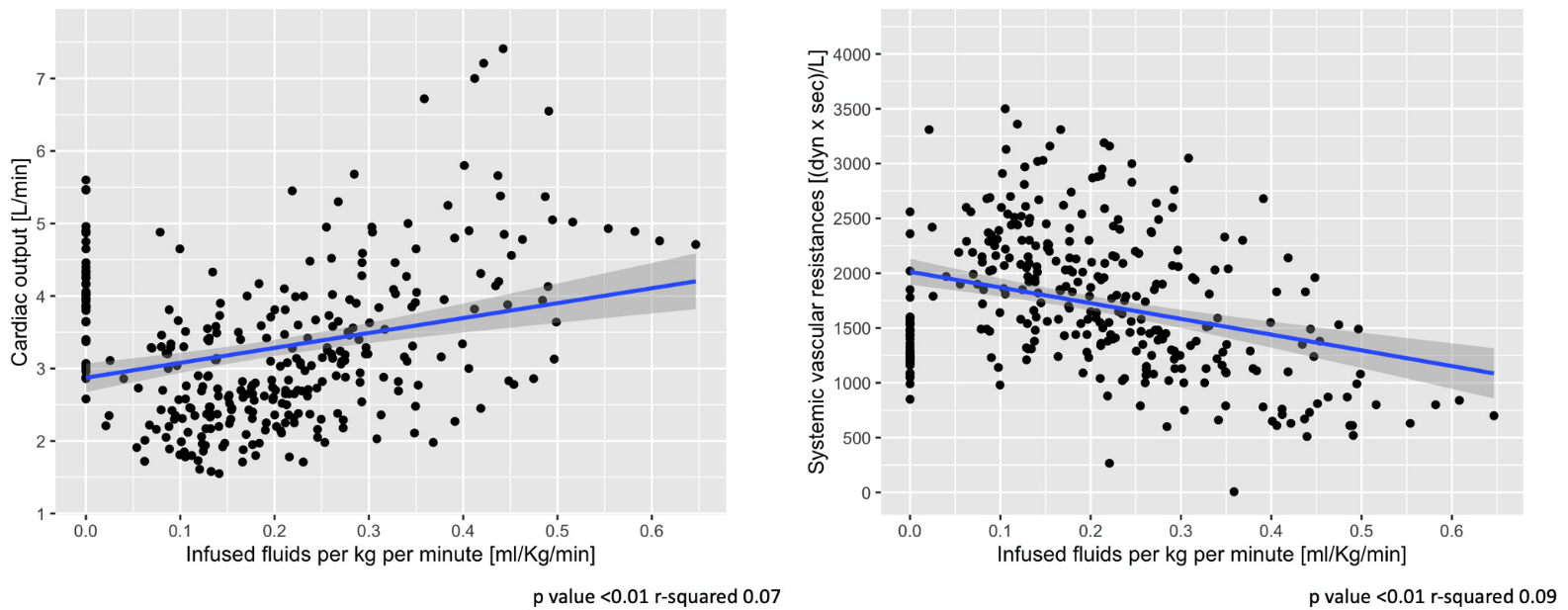


Figure S4: Association between cardiac output and vascular resistances vs. micrograms of infused noradrenaline


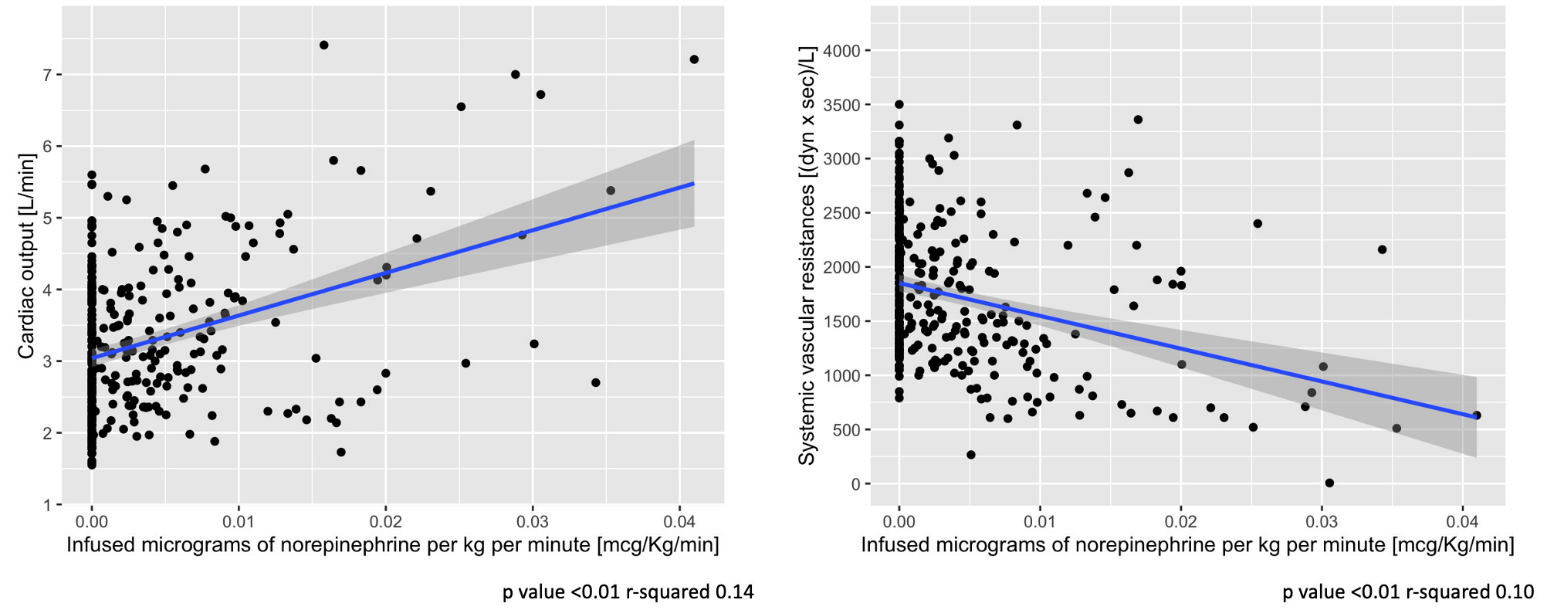


Figure S5: Variation of ECV and ICV according to the shifted amount of fluids





Figure S6: End-of-experiment association between lungs weight and wet-to-dry ratio vs. pleural pressure





Figure S7: Association between the infused fluids and norepinephrine vs. systolic arterial pressure.





**Sodium distribution in a second cohort of animals:**

Twelve pigs were enrolled in a study investigating the physiologic responses to three infusion fluids: normal saline, Ringer's lactate solution and soluzione reidratante III GS (1L composition: Na^+^ 140 mmol, K^+^ 10 mmol, Ca^++^ 5 mmol, Mg^++^ 3 mmol, Cl^-^ 103 mmol, Acetate as HCO3^-^ 47 mmol, Citrate 8 mmol. Theoretical osmolarity: 307 **mOsm/L, pH 5.0-7.0). All the animals received a 6h long infusion of each one of the three fluids; between infusions there was a time-interval of one hour. Thus, the total duration of the experiment was 20 hours. The sequence of the infused fluids was randomized.**

We had access to all data regarding the weight of the animals, the amount of infused and eliminated fluids and sodium and bloodwork analyses. Analyses were performed at the following times: 0, 6, 7, 13, 14 and 20h. Plasma osmolarity was not measured so we calculated it with the following formula: [Osm] = 2 x ([Na^+^] + [K^+^]) + Glucemia/18 + Urea/6.

We assessed sodium distribution using the same approach we used in our 78 animals; we calculated: 1. baseline extracellular and intracellular volumes (respectively 20 and 40% of the total body weight); 2. theoretical sodium concentration and osmolarity, fluid shift and the new values of extracellular and intracellular volumes, before and after the osmotic equilibrium; 3. we assessed the difference between the calculated osmolarity with the standard formula ([Osm] = 2 x ([Na^+^] + [K^+^]) + Glucemia/18 + Urea/6] and with the two-compartment model; 4. we estimated the amount of missing sodium with the following formula:

Na_miss_ = [Na]_bas_ x ECV_bas_ + Na_ret_ - [Na^+^]_act_ x (ECV_bas_ + V_ret_ + V_shift_)

At the end of the experiment the actual sodium concentration was 141.6 mmol/L while the calculated sodium concentration after osmotic equilibrium was 150.3 mmol/L (see figure A). Osmolarity with the standard formula was 298.4 while the osmolarity computed with the two-compartment model was 309.0 (figure B).

Baseline intracellular and extracellular volumes were: 8.85 and 4.42 L, while at the end-experiment we calculated the following intracellular and extracellular volumes: 8.50 and 6.89 L (figure C). Out of a net retained sodium of 412.9 mmol, the amount of missing sodium was 56.0 mmol (13.6%).

Figure A: Measured and calculated sodium concentration throughout the experiment





Figure B: Calculated osmolarity with the standard formula and with the two-compartment model





Figure C: Extracellular and intracellular volumes throughout the experiment
